# Peripheral Nerve Repair with Cultured Schwann Cells: Getting Closer to the Clinics

**DOI:** 10.1100/2012/413091

**Published:** 2012-06-04

**Authors:** Maria Carolina O. Rodrigues, Antonio Antunes Rodrigues, Loren E. Glover, Julio Voltarelli, Cesario V. Borlongan

**Affiliations:** ^1^Center of Excellence for Aging and Brain Repair, Department of Neurosurgery and Brain Repair, University of South Florida College of Medicine, 12901 Bruce B. Downs Boulvard, Tampa, FL 33612, USA; ^2^Department of Internal Medicine, Faculty of Medicine of Ribeirão Preto, University of São Paulo, 14000 Ribeirão Preto, SP, Brazil; ^3^Department of Surgery and Anatomy, Faculty of Medicine of Ribeirão Preto, University of São Paulo, 14000 Ribeirão Preto, SP, Brazil

## Abstract

Peripheral nerve injuries are a frequent and disabling condition, which affects 13 to 23 per 100.000 persons each year. Severe cases, with structural disruption of the nerve, are associated with poor functional recovery. The experimental treatment using nerve grafts to replace damaged or shortened axons is limited by technical difficulties, invasiveness, and mediocre results. Other therapeutic choices include the adjunctive application of cultured Schwann cells and nerve conduits to guide axonal growth. The bone marrow is a rich source of mesenchymal cells, which can be differentiated *in vitro* into Schwann cells and subsequently engrafted into the damaged nerve. Alternatively, undifferentiated bone marrow mesenchymal cells can be associated with nerve conduits and afterward transplanted. Experimental studies provide evidence of functional, histological, and electromyographical improvement following transplantation of bone-marrow-derived cells in animal models of peripheral nerve injury. This paper focuses on this new therapeutic approach highlighting its direct translational and clinical utility in promoting regeneration of not only acute but perhaps also chronic cases of peripheral nerve damage.

## 1. Introduction

 The incidence of peripheral nerve injury in developed countries is estimated between 13 and 23 per 100.000 persons per year [1–3]. Common reported causes include motor vehicle accidents, lacerations with blades, glass or long bone fractures, and sports injuries. These statistics may be underestimated since only traumatic injuries that reach the health care system are included. Nontraumatic injuries, nerve damage secondary to abdominal or pelvic surgeries, and lesions that are not treated at a health facility remain to be accounted [3]. As an example, erectile dysfunction secondary to prostate surgeries associated with iatrogenic transection of erectile nerves occurs in approximately 35 to 86% of radical prostatectomy surgeries [4, 5]. It is, however, in the war scenario where the problem is more frequent. Blast injuries predominate, often associated with complex soft tissue and arterial wounds, most of which requiring limb amputation [6].

 Peripheral nerve injuries are classified according to their severity, from type I, limited to demyelination, through type V, which comprises disruption of all three layers of the nerve endoneurium, perineurium, and epineurium. A sixth type of injury has also been described, with different degrees of lesion within individual fascicules from the same nerve [6]. Recovery is inversely proportional to the severity of damage. Endogenous repair initiates after injury but usually does not sustain itself beyond 12 months [7]. If the nerve does not heal within this period of time, recovery is unlikely and chronic pain and disability may persist. In serious injuries, surgical intervention is usually necessary, aiming to align the damaged axonal stumps, either through end-to-end sutures or by interposition of a nerve graft or even a scaffold for axonal growth. Frequently, however, the procedure does not suffice, and functional recovery is suboptimal [2, 6, 7].

 Nerve repair is often disappointing and, despite best efforts, full functional recovery is seldom achieved, especially regarding motor nerves [2]. The high incidence of peripheral nerve damage, associated with poor reparative outcomes, indicates the importance of the problem and the demand for better regenerative therapies. In this context, the development of new approaches, including cell-based therapies, may improve outcomes and diminish the resulting disability of affected victims. Indeed, preclinical ongoing studies already show positive results in models of erectile, facial, optic, and sciatic nerve injuries [5, 8–11].

## 2. The Healing Process That Follows Nerve Injury

 Peripheral nerve injury is followed by responses both from segments of the axon, proximal, and distal to the damaged area, and from the surrounding neural and nonneural cells. As a first endogenous reparative attempt, the neuronal cell body increases in size, the Nissl bodies dissolve, and the nucleus migrates peripherally, initiating a process of protein synthesis [12]. The distal detached nerve undergoes intrinsic fragmentation, as the first stage of Wallerian degeneration. Interestingly, distal nerve disintegration does not start immediately upon injury. In primates, axons remain intact during days and can still transmit electric potentials when stimulated [13, 14]. The following fragmentation of the axon is a rapid event and seems to be mediated by activated intrinsic proteases, including calpain and the ubiquitin-protease system, and once started, it is completed within hours [15–17]. Axonal and myelin debris is later phagocytosed by local Schwann cells and recruited macrophages [18]. These latter cells opportunely infiltrate through the broken blood-nerve-barrier and myelin sheathing, attracted by specific chemokines and cytokines [12, 19]. Neutralization of these factors by specific antibodies delays demyelination and, in consequence, axonal regeneration [12]. In fact, clearance of myelin debris seems to be an important step for repair since myelin contains inhibitors of axonal growth and may, therefore, impair regeneration [20].

 Schwann cells have a central role in nerve repair, not only concerning Wallerian degeneration and remyelination but also in promoting axonal growth. Experiments in which these cells were either decreased in number or made nonfunctional indicate a deficient axonal regeneration, placing the Schwann cell as an integral component of the regenerative process [21]. After injury, the Schwann cells become activated, assume a more primitive phenotype, and stimulate axonal growth, with upregulation of growth-related genes, including those that encode intrinsic neurotrophic factors and key transcription factors [2, 8, 22]. Activated Schwann cells produce collagen and laminin, creating a tunnel of extracellular matrix, and express cell adhesion molecules and receptors, including IL-1, N-cadherin, gamma integrins, and the neural cell adhesion molecule (N-CAM) [2].

 The mechanisms through which Schwann cells and the damaged axons communicate are poorly understood. Vrbova et al. suggested that an interaction between neurotransmitters and their upregulated receptors may be involved and demonstrated that drugs that inhibit the binding of acetylcholine to its receptors and the binding of ATP to P2Y receptors decrease axonal regeneration [23]. Additionally, HNK-1 glycan, which is a marker of regeneration mediated by Schwann cells in motor nerves, was decreased in the animals with neurotransmitter blockage [23, 24]. Goethals et al. describe Toll-like receptors as mediators of damage recognition between Schwann cells and axons [25]. Schwann cells constitutively express Toll-like receptors and, therefore, play a sentinel role. Toll-like receptor ligands released by degenerating axons stimulate Schwann and immune cells to clear myelin and start the reparative process.

 The resulting supportive environment yields Schwann cell proliferation, formation of Büngner bands, and, finally, axonal sprouting [2, 26, 27]. This sprouting occurs at a rate of 1 to 4 mm per day, with progressive myelination of the fibers by the neighboring Schwann cells. The final repaired nerve usually presents thinner myelin sheets with shorter nodal lengths, less functional than the original nerve [28]. Time is an important factor affecting nerve repair. The supportive response mediated by neural and nonneural cells is more pronounced in the few months that follow nerve injury [6, 7, 28, 29]. When surgical intervention is delayed, the chronically denervated Schwann cells begin spontaneous apoptosis and repair becomes less probable. Recently, Gordon et al. demonstrated that although denervation of the distal stump is the main determinant, the denervated muscle also negatively impacts functional outcome [29]. Therefore, attempts to enhance nerve regeneration should be initiated as early as possible.

## 3. Interventions to Improve Peripheral Nerve Repair

 Nerve repair requires a complex interaction of a scaffold for axonal proliferation, supportive cells such as the Schwann cells, growth factors, and extracellular matrix [2, 30]. Therefore, alignment of the nerve stumps stands as one of the first interventions after peripheral nerve injury. When end-to-end suture of the nerve is not possible, the interposition of a nerve conduit becomes necessary. Autologous grafts are often harvested from the sural or sensory nerves; however, sometimes there are limitations such as tissue availability, size incompatibilities, and deformities. Nevertheless, autologous nerve grafts are considered the gold standard for repairing peripheral nerve gaps [31]. Less frequently, allografts can be used, with the disadvantages of requiring immunosuppression and of producing worse outcomes than autografts.

 More recently, tissue engineering has provided nerve conduits, which function as guides for axonal growth. Depending on the materials used for their construction, they can be classified as natural, when based on laminin, collagen or even vessels and decellularized nerves, or artificial, usually made of polymers. Artificial, nonbiodegradable scaffolds aid nerve growth and provide beneficial results. However, these conduits may lead to chronic inflammation and tissue compression and therefore must be surgically removed once neural healing has been concluded. To avoid the hurdles of a second surgery on the injury site, biodegradable scaffolds are preferred.  Table 1 includes some examples of experimental studies that used biodegradable scaffolds. Concerning humans, recently Åberg et al. suggested that wrapping-around injured wrist/forearm nerves with a resorbable poly[(R)-3-hydroxybutyrate] (PHB) implant, promoted significant sensory and motor improvements when compared to end-to-end epineural suture alone [32]. Importantly, no adverse effects were associated with the procedure, therefore considered safe.

Sensory nerves usually have better outcomes than motor nerves, after reconstructive interventions [48]. However, sensory repair is only warranted in areas of critical sensation, while motor nerves are usually indispensable. Motor nerve repair is mostly worth when the site of injury is close to the end organ because if the length to be repaired is too long, by the time reinnervation is achieved, the end-organ may be already unresponsive. In these cases, nerve transfer procedures, if available, may be attractive alternatives.

 The major limitation for use of nerve conduits is the low rate of axonal growth, which may not yield full repair within the available time. Studies have shown that the conduits are effective in promoting repair of nerve gaps measuring up to 3 cm, while nerve autografts are required for bridging of larger distances [49, 50]. As an attempt to apply nerve guides for axonal growth in larger nerve injuries, Kokai et al. used polymer tube-like scaffolds associated with microparticles of GDNF, aiming to increase the rate of axonal growth and improve the success of functional restoration [40]. Endogenous Schwann cells have been shown to be attracted by the GDNF particles, increasing the rate of nerve regeneration. Similarly, Liu et al. observed higher nerve repair rates in rats with peripheral nerve injury treated with biodegradable conduits associated with nerve growth factor (NGF) [51].

 Schwann cells are also potentiated by associations with other cell types or by bioengineering. Olfactory ensheathing cells (OECs) successfully repair peripheral nerve injuries and with less efficiency central nervous system axonal lesions [52]. However, Verdú et al. and You et al. demonstrated that the association of Schwann cells and OECs promotes better myelination and functional repair to injured sciatic axons than either cell type alone [53, 54]. Another studied approach involves genetic engineering, inducing the Schwann cells to express cell adhesion molecules, therefore improving their regenerative capacity. Increasing the rate of axonal repair may enhance the chances of successful functional recovery since the outcomes are in most cases time dependent. Lavdas et al. have investigated the effects of Schwann cells genetically modified to overexpress cell adhesion molecules, mostly the polysialylated form of neural cell adhesion molecule NCAM, on nervous system repair [55, 56]. It is hypothesized that these adhesion molecules improve transplanted cell migration, increasing their interaction with the host environment, therefore promoting more efficient functional integration. Additionally, cell adhesion molecules also stimulate axonal growth and remyelination, increasing the rate of renervation [57, 58].

## 4. Sources of Schwann Cells and Experimental Studies

 Schwann cells are embryologically derived from the neural crest [59, 60]. Some neural crest pluripotent and stem cells (NCSCs) persist in adult specimens in sites of gliogenesis such as the sciatic nerve and dorsal root ganglia. Migration, proliferation, expression of growth factors, and remyelination capacity are listed features associated with Schwann cells, indicating their stem cell behavior [61, 62]. Attempts to use Schwann cells expanded in culture for neural repair have been described in the literature for over two decades [62]. Recently, Hood et al. used artificial tubes filled with autologous Schwann cells to replace gaps between sciatic nerve stumps of rats, with reported functional recovery [63].

 The combination of axonal scaffolds and transplanted Schwann cells provides adequate support for peripheral nerve repair and has been investigated in the experimental setting as a strategy to circumvent the limitations of surgical repair [34, 37, 45]. The cells can be delivered inside a hollow tube, seeded in a hydrogel and subsequently filled into the lumen of the conduit, or placed inside a conduit containing a supportive structure [30]. Most studies use the first type of delivery, but it may be associated with substantial cell loss [38, 43, 46, 63]. Improved outcomes have been obtained using the second [34, 37] and third [45] approaches, but these are more technically complicated and costly. Although successful, these procedures are limited by the high number of cells required for each intervention, potential injury to the donor nerve, and fibroblast contamination of the cultures [64]. Therefore, alternative sources of supportive cells have been researched.

 The bone marrow may be an attractive source despite the large number of available tissues from which Schwann cells can be obtained. Bone marrow mesenchymal cells have been studied for years in the context of *in vitro* expansion and transplantation [65]. Their ease of access, abundance, and safety are important attributes, especially concerning clinical application. Mesenchymal stromal cells (MSCs) or bone marrow stromal cells are multipotent somatic stem cells, from a nonhematopoietic precursor anchored in bone marrow that can differentiate in mesodermal cell linages, neural phenotypes, and also into Schwann-like cells [47, 66]. When MSCs are placed in an appropriate culture media, they transdifferentiate into a glial cell phenotype that expresses S100, GFAP, and p75 [67, 68]. *In vitro* differentiated cells show promising results when associated with artificial conduits and acellular grafts, promoting axonal and functional recovery [39, 41, 67].

Table 1 describes several preclinical studies that evaluate the reparative effects of different kinds of cell therapy upon peripheral nerve damage. Besides nervous tissue and bone marrow, other alternative sources of cell are investigated, such as the adipose tissue and skin. The skin dermis is another investigated cell source and contains neural crest-related precursors skin precursor cells SKPCs that can differentiate into neural crest cell types *in vitro*, including those with characteristics of peripheral neurons and Schwann cells [33, 69]. Also, the bulge area of the hair and whisker follicles harbors NCSC that can differentiate into Schwann-like cells, becoming an accessible source of cells for transplant. Both NCSC and SKPC have been used in experiments of peripheral nerve and spinal cord injuries with functional improvement after transplantation [33, 42, 70, 71].

 Regarding the adipose tissue, Reid et al. showed that differentiated adipose-derived Schwann cells expressed a range of intrinsic neurotrophic factors [35, 72]. They also demonstrated that the incorporation of either adipocyte-derived or nerve-derived Schwann cells into the repair site significantly increased the expression of anti-apoptotic m-RNA of Bcl-2 and decreased proapoptotic m-RNA Bax and caspase-3 in dorsal root ganglia, therefore promoting a neuroprotective effect [72]. In a comparative study for sciatic nerve repair using a fibrin conduit seeded with different cell populations, primary Schwann cells showed better results over adipose and bone-marrow-derived mesenchymal stem cells; however, the two last cell types still showed better axonal regeneration than the control, nontreated group [36]. Adipose-tissue-derived Schwann cells have also been used in experimental models of facial palsy with positive results [44]. Sun et al. published a study in rats with lesions of facial nerve that presented functional and electrophysiological recovery of nerve injuries measuring 0.8 cm. 

 Neural stem cells directly transplanted in the site of peripheral nerve injury differentiate into Schwann-like cells, secrete neural growth factors, and improve nerve regeneration [8]. In addition, these cells can enhance compatibility between transplanted and surrounding tissue, decreasing the necessity of immunosuppressive agents. Moreover, the coculture and cotransplantation of neural stem cells with Schwann cells increase the differentiation of neural stem cells into neurons and improve functional recovery in models of spinal cord injury [73].

 Recently, Wakao et al. used collagen nerve conduits filled with autologous bone marrow MSC differentiated *in vitro* into Schwann cells to promote repair of median nerve injuries in monkeys [47] (Table 1). Successful outcomes were reported, with functional, histological, and electromyographical improvements. Interestingly, Hu et al. demonstrated that allogeneic acellular nerves filled with autologous, but nondifferentiated, bone-marrow-derived MSC also promoted functional recovery when placed in ulnar nerve gaps of nonhuman primates [31]. The success rates were comparable to those from nerve autografts and autologous Schwann-cell-filled acellular nerves and better than controls, which received the acellular nerves not filled with cells. The similar outcomes of both studies raise the hypothesis that predifferentiation of MSC into Schwann cells may not be necessary to achieve repair. Moreover, a previous study had demonstrated that allogeneic Schwann cells locally transplanted into a rat model of spinal cord injury induced the infiltration of endogenous Schwann cells into the damaged site [74]. Although the observation should not be transposed to peripheral nerve injury, it suggests a paracrine, rather than replacement therapeutic effect. On the other hand, Sun et al. recently published a study in rats with facial nerve injuries treated with decellularized allogeneic arteries filled with adipose-tissue-derived Schwann cells, where transplanted cells survived in the graft over 8 weeks [75]. Studies establishing the exact fate of labeled Schwann cells are still needed. Although *in vitro* studies demonstrate differentiation of the MSC into different cell types, mainly of mesodermal lineage, *in vivo* studies fail to show convincing evidence of such event. It is possible that the MSCs promote healing effects mostly through paracrine action, inducing recruitment of neighboring host cells, modulation of local and perhaps systemic immune response, and improving cell survival, than through direct cell differentiation.

## 5. Clinical Trial of Nerve Grafts and Scaffolds

 Clinical applications of cell therapy for peripheral nerve repair are rare. A clinical trial conducted by the University of Stanford used scaffolding tubes filled with longitudinal collagen fibers as guides for axonal growth [76]. Such tubes were occupied by autologous cultured Schwann cells and served as a replacement for nerve autografts, aiming to repair short and long gaps. Results were partially reported, including cell harvesting, expansion in culture, and assembly of cells and nerve conduits, but clinical outcomes have not yet been published [76]. Another proposed study (ClinicalTrials.gov identifier: NCT00953277), currently recruiting patients, intends to evaluate the effects of a commercial nerve scaffold on erectile function and continence after video laparoscopic radical prostate excision [77]. The surgery is associated with high incidence of erectile nerve transection and directly affects postoperative quality of life of the patients [4, 5]. According to advertisements, the product is derived from human decellularized nerves and may stand as an “off the shelf” product, viable for three years in frozen storage [78]. The same company sponsors a comparative study between the above-mentioned commercial human-derived nerve scaffold and synthetic or biosynthetic hollow tubes to repair peripheral nerve gaps in the hand (ClinicalTrials.gov identifier: NCT00948025) [79]. Although not yet approached by these last two studies, cell application to the nerve conduits may be attempted in the future.

## 6. Practical Issues

**Figure 1:**
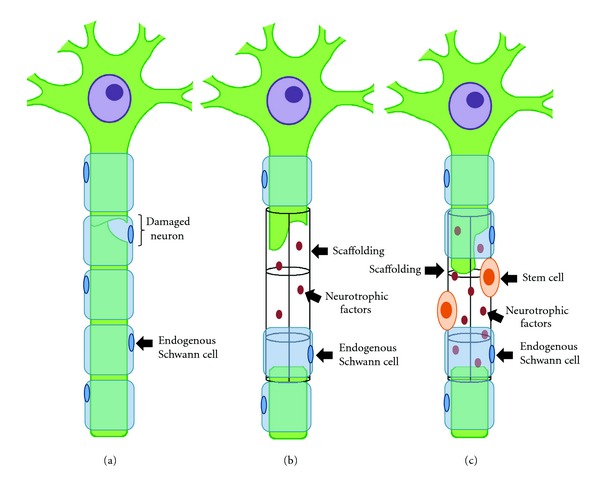
Stem cell with scaffold support in peripheral nerve injury. (a) Nerve injury with stem cell transplantation shows limited remyelination and axonal growth between the proximal and distal stumps. (b) Nerve injury with scaffolding devices can be fabricated to secrete neurotrophic factors aiming to enhance the regenerative process, but still the outcome results in limited remyelination and axonal growth between the proximal and distal stumps. (c) The combined use of stem cells and scaffolding promotes best results, allowing an improved substrate for cell-to-cell contact and increased concentrations of released neurotrophic factors. Additionally, transplanted stem cells may stimulate endogenous Schwann cells to proliferate at a higher rate, thus enhancing remyelination and axonal growth following peripheral nerve damage.

 Cell-based therapy associated with scaffolds is a promising branch of regenerative medicine. Nerve injuries are a frequent and disabling condition, usually affecting young and productive subjects, which may benefit from such procedures. The idea of using nerve conduits filled with bone-marrow mesenchymal cells may be an attractive alternative to more aggressive therapies. If successful, the treatment may lead to functional improvement, avoiding the hurdles of additional surgeries and use of immunosuppressive drugs. A hypothetical scenario would be a patient with a traumatic limb injury, including extensive and severe damage of a motor nerve, resulting in acute disability. There would be a nerve gap, precluding any attempt of direct nerve suture. The use of a nerve conduit filled with allogeneic bone marrow-derived MSCs would be proposed, as an alternative to the standard autologous nerve graft implant. The acute nature of the condition would require a readily available source of cells, and since MSCs are immunoprivileged, indicating poor alloimmune response and therefore delayed rejection, allogeneic sources would be ideal. The cells would be already isolated from donated bone marrow, expanded in culture without further manipulation, tested for safety and quality, cryopreserved and ready for clinical use. The necessary amount of cells would then be thawed and inserted into biodegradable nerve conduits, readily implanted between nerve stumps during microsurgery. The therapy would require no immunosuppression and sequential functional and electromyographical evaluations would determine the outcomes. Expected results would be axonal repair, remyelination, and progressive functional improvement, either through differentiation of the transplanted into Schwann-like cells or, most probably, through paracrine effects of the bone-marrow-derived MSCs on the proximal axonal stump and remaining endogenous Schwann cells, stimulating regeneration (see Figure 1).

 Cell banking is a developing resource for repair in degenerative, inflammatory, and traumatic disorders. The rising number of cell banking companies reflects the increasing number of emergent therapeutic applications for cells derived from the bone marrow, cord blood, and multiple other tissues. Although the regenerative potential and the properties of the cells change slightly from tissue to tissue, the bone marrow still stands as a preferred source of hematopoietic and mesenchymal cells, due safety, easy harvesting, and previous clinical experience [65]. Allogeneic cell banking seems more logical as an “off the shelf” product, however investments in autologous cell preservation may be also interesting, as an insurance against future illnesses. Transplanted autologous MSCs may persist in the injury site longer because of absent immune rejection, therefore promoting long-lasting effects.

 The application of MSCs is associated with some hurdles. The most obvious is cell viability after thawing and insertion into the conduit. Orthopedic reconstructive surgeries are often long-lasting and the exposure of the cells to a hostile environment may jeopardize their survival. Therefore, one approach would be to leave cell implantation as a final step of the surgery, immediately followed by closure of the incision. Perhaps, performance of the surgery by a skilled surgeon may be critical. Additional aggressions to the cells would be the presence of infection or any situation leading to an acidic or hypooxygenated environment. Therefore, these conditions must be strictly controlled before cell transplantation. A second concern is that MSCs may lead to abnormal, nonlinear nerve growth, due to aberrant stimulation of the sprouting axonal buds. Although the problem has been observed in nerves stimulated with growth factors [80], such behavior is not expected following MSC grafts. This potential problem may be prevented through use of good quality conduits, possibly containing an internal supportive structure, aiming to guide newly formed axons in the direction of the distal nerve stump. Third, the therapy may promote cell differentiation toward a nonexpected cell type. Malignancy is not reported as a usual consequence of MSC administrations, but it is possible that due to the local host conditions the cells differentiate into nonneural cell types. Another concern that may be discussed is the functional integration of the transplanted cells into the injured nerve [81], although repair of peripheral nerve is usually more successful than central nervous system injuries. Finally, some donor conditions may interfere with MSC cultures, such as advanced age, smoking, and diabetes [82, 83]. In these cases, autologous use may not be the best choice.

 In summary, despite the reported therapeutic benefits of MSC therapy in experimental models of peripheral nerve injury, there remain translational hurdles that still need to be addressed before the realization of large clinical trials for this treatment. In the future, this form of cell therapy may aggregate more complex features, such as addition of trophic factors within the conduits, manipulation of the cells for expression of neurotrophic factors or even adhesion molecules. On the other hand, the procedure may remain simple and enable the investigation of therapeutic pathways, perhaps unveiling a specific substance that would be therapeutically active, therefore sparing the use of cells.

## 7. Conclusions

 Spontaneous peripheral nerve repair is disappointing even after microsurgical intervention, especially concerning severe injuries. Resulting chronic pain and disability affects victims of all ages, including those in economically active phase of life [3]. The available treatment using nerve grafts is very limited and invasive, and new therapeutic options are required. Schwann cell cultures have demonstrated favorable results in the experimental setting; however, the ideal source of cells has not yet been established. Bone-marrow-derived mesenchymal cells present encouraging results and may become the ideal cell for clinical translation. These cells have been exhaustively investigated during the last two decades and approved for use in numerous clinical trials, especially aiming at immunomodulation and tissue repair [84].

The bulk of preclinical studies offer many advantages of MSC therapy for peripheral nerve injury. The first advantage of bone-marrow-derived MSC is the uncomplicated and minimally invasive harvesting of the cells through aspiration of reduced volume of bone marrow from the iliac crest. Second, *in vitro* cultures are in most cases simple, with adequate proliferation rates and high cell viability [65, 66]. Large number of cells can be obtained within a few weeks. Third, these cells are considered safe, and not associated with malignancy, uncontrolled proliferation or graft-versus-host disease. Fourth, MSCs are immunologically privileged and, thus, even allogeneic cells are not readily rejected by the host. Although some immune response is elicited through time, the cells survive long enough for therapeutic effectiveness without need of immunosuppressant drugs [84]. Fifth, *in vitro* differentiation into Schwann cells is possible and already described by several authors [30, 37, 63]. Finally, MSCs secrete trophic factors, stimulating neuron regeneration, and, possibly, contributing to a supportive environment [85].

 Cultured cells, combined with adjunctive treatments such as axonal growth guides, may be a feasible approach for future clinical studies (see Figure 1). Severe injuries in which nerve grafts are not available may benefit from this strategy that may provide an extracellular substrate for the transplanted cells and spared host cells allowing a bridge for cellular integration and/or a cellular and molecular pathway for improved paracrine effects. Moreover, studies investigating the effects of these cells over situations of delayed surgical intervention and chronic axonal denervation have not been reported. The neurotropic effect of these cells, combined with a favorable effect over the surrounding environment, may prolong the time window for axonal regeneration and, perhaps, improve the rates of functional restoration even in chronic cases.

## Figures and Tables

**Table 1:** Preclinical studies evaluating cell therapy for peripheral nerve repair*.

Study	Injury type/animal model	Cellular type/factor	Major findings
McKenzie et al. [33]	Sciatic nerve crush injury in myelin-deficient mice	Skin-derived precursors differentiated into Schwann cells	Remyelination and functional recovery
Udina et al. [34]	Sciatic nerve injury in mice (0.6 cm gap)	Collagen guides seeded with allogeneic Schwann cells plus FK-506	Successful regeneration and functional recovery
Negishi et al. [11]	Optic nerve injury in rats (transection)	Extracellular matrix Schwann cells and neurotrophins	Axonal regeneration of retinal ganglion cells
Reid et al. [35]	Sciatic nerve injury in rats (1.0 cm gap)	Adipose-derived stem cells	Dorsal root ganglia protection from apoptosis
di Summa et al. [36]	Sciatic nerve injury in rats (1.0 cm gap)	Nerve fibrin conduits seeded with adipose-derived stem cells	Enhanced peripheral nerve repair
Evans et al. [37]	Sciatic nerve injury in rats (1.2 cm gap)	Biosynthetic conduits seeded with Schwann cells	Increased nerve regeneration
Koshimune et al. [38]	Sciatic nerve injury in rats (1.2 cm gap)	Bioabsorbable Schwann cell-coated conduits	Axonal regeneration
Ladak et al. [39]	Sciatic nerve injury in rats (1.2 cm gap)	Bone marrow MSCs differentiated into Schwann-like cells seeded in collagen conduits	Regeneration of sciatic motoneuron
Kokai et al. [40]	Sciatic nerve injury in rats (1.5 cm gap)	Scaffolds containing GDNF microparticles	Increased rate of nerve regeneration; migration and proliferation of Schwann cells
Dezawa et al. [41]	Sciatic nerve injury in rats (1.5 cm gap)	Bone marrow MSCs differentiated into Schwann-like cells suspended in Matrigel injected into hollow fibers	Successful nerve regeneration and myelination
Marchesi et al. [42]	Sciatic nerve injury in rats (1.6 cm gap)	Guides filled with skin-derived stem cells	Functional recovery and myelination
Ansselin et al. [43]	Sciatic nerve injury in rats (1.8 cm gap)	Nerve guides filled with syngeneic Schwann cells	Successful nerve regeneration conditional to number of cells implanted
May et al. [5]	Cavernous nerves sections in rats (0.5 cm gap)	Silicon tubes seeded with GDNF-transduced Schwann cells	Increased recovery of erectile function
Sun et al. [44]	Facial nerve injury in rats (0.8 cm gap)	Decellularized artery allografts with autologous adipose-derived stem cells	Nerve repair and functional recovery
Wang et al. [10]	Facial nerve injury in rabbits (1.0 cm gap)	Autologous vein graft filled with autologous MSCs differentiated into Schwann cells	Improvement of functional recovery and upregulated myelin mRNA
Cheng and Chen [45]	Sciatic nerve injury in rabbits (2.0 cm gap)	Polyglactin scaffolds seeded with Schwann cells and coated with biomembranes	Successful nerve regeneration
Zhang et al. [46]	Tibial nerve injury in rabbits (4.0 cm gap)	Autogenous venous graft filled with Schwann cells	Successful nerve regeneration and electromyographic improvement
Wakao et al. [47]	Non-human primates median nerve injury (2.0 cm gap)	Collagen guides seeded with bone marrow MSC-derived Schwann cells	Functional, histological, and electromyographical recovery
Hu et al. [31]	Non-human primates ulnar nerve injury (4.0 cm gap)	Acellular allogeneic nerve grafts with autologous MSCs	Structural and functional peripheral nerve repair

* The studies are grouped by animal model, nerve type, and injury size, starting with mice, followed by rats, rabbits, and nonhuman primates. The table does not list all the available studies but describes the main publications.
